# Nutrition Assessment and Management in Patients with Cirrhosis and Cognitive Impairment: A Comprehensive Review of Literature

**DOI:** 10.3390/jcm11102842

**Published:** 2022-05-18

**Authors:** Jessica Faccioli, Silvia Nardelli, Stefania Gioia, Oliviero Riggio, Lorenzo Ridola

**Affiliations:** Department of Translational and Precision Medicine, Sapienza University of Rome, 00185 Rome, Italy; jessica.faccioli@uniroma1.it (J.F.); nardelli.silvia@gmail.com (S.N.); stensgioia@hotmail.com (S.G.); oliviero.riggio@uniroma1.it (O.R.)

**Keywords:** hepatic encephalopathy, protein caloric-malnutrition, sarcopenia, dietary intervention, cirrhosis, mortality

## Abstract

Hepatic encephalopathy (HE) represents a common complication of liver cirrhosis. Protein-calorie malnutrition is frequently encountered in the cirrhotic patient and its most obvious clinical manifestation is sarcopenia. This condition represents a risk factor for HE occurrence because skeletal muscle acts as an alternative site for ammonium detoxification. Preventive intervention through an adequate assessment of nutritional status should be carried out at early stages of the disease and in a multidisciplinary team using both non-instrumental methods (food diary, anthropometric measurements, blood chemistry tests) and instrumental methods (bioimpedance testing, DEXA, CT, indirect calorimetry, dynamometry). Dietary recommendations for patients with HE do not differ from those for cirrhotic patient without HE. Daily caloric intake in the non-obese patient should be 30–40 Kcal/Kg/day with a protein intake of 1–1.5 g/Kg/day, especially of vegetable origin, through 4–6 meals daily. In patients with HE, it is also essential to monitor electrolyte balance, supplementing any micronutrient deficiencies such as sodium and zinc, as well as vitamin deficiencies because they can cause neurological symptoms similar to those of HE. In light of the critical role of nutritional status, this aspect should not be underestimated and should be included in the diagnostic–therapeutic algorithm of patients with HE.

## 1. Introduction: Hepatic Encephalopathy

Hepatica encephalopathy (HE) represents one of the most frequent complications of liver cirrhosis and certainly one of the most debilitating, with a negative impact on quality of life, morbidity and mortality. 

It affects 30–45% of patients with liver cirrhosis and up to 50% of patients with TIPS. It is characterized by an altered cognitive status and neuromuscular function, and its onset depends on the degree of hepatocellular failure and the presence of porto-systemic shunts. 

HE must be qualified by the context in which it occurs, severity, precipitating factors and response to treatments.

Regarding the severity of the episode, the new classification suggested by the AASLD/EASL guidelines identify two forms of HE and introduces the term “overt” to refer to asymptomatic or only mildly symptomatic patients.

In particular, “covert” EE includes two forms, “minimal” HE (MHE) and HE of grade I according to West Haven criteria; patients with MHE, despite the absence of clinical evidence of abnormalities in cognitive status, present neuropsychological alterations in tests that explore psychomotor speed and cognitive functions.

Patients with grade I HE, despite normal spatiotemporal orientation, present some degree of cognitive/behavioural impairment compared to normal habits.

The “overt” HE (OHE) includes patients with temporo-spatial disorientation, inappropriate behaviours, agitation or drowsy up to coma [1,2].

Sleep disturbances are common in cirrhotic patients with a prevalence varying between 27 to 70%. The most common sleep disorders are: sleep onset insomnia, fragmented sleep, difficulty falling asleep after nocturnal awakenings, shortened sleep duration and poor sleep quality. 

Patients with HE may have hypersomnia, somnolence, excessive and inappropriately timed sleepiness and long daytime naps up to sleep–wake inversion in severe OHE. A study by Singh et al. showed that excessive day time sleepiness and impaired sleep quality were common in patients with MHE and correlated with neuropsychiatric impairment; in addition, the improvement of MHE with lactulose also lead to improvement in sleep disturbances and HRQOL [3].

The pathogenesis is multifactorial. It has been shown that hyperammonaemia, through adenosine, correlates with daytime sleepiness and disruption of sleep architecture. Furthermore, abnormalities in the circadian rhythm of melatonin of both central and peripheral origin may play a role. Finally, disturbances in the 24-h rhythm of skin temperature have been recently reported in patients with cirrhosis, with impaired thermoregulation.

Sleep disturbances can be assessed with diaries and questionnaires, polysomnography and actigraphy [4].

The diagnosis of HE is a challenge for the clinician. Difficulties arise primarily in the presence of “covert” forms of HE or in cases where there are no true precipitating factors. 

Precipitating factors include classic ones such as infections, digestive hemorrhage, medications, constipation, dehydration and electrolyte imbalances and “new precipitants” such as sarcopenia and spontaneous or iatrogenic porto-systemic shunts.

The diagnosis of OHE is generally a diagnosis of exclusion and is based primarily on the objective examination and judgment of the clinician. The approach that can be used is the “two-step” approach. 

The first step is to determine whether the patient has a known history of hepatopathy and whether the latter is severe enough to warrant an episode of OHE. 

The second step is to rule out other causes of neuro-psychiatric symptoms such as acute alcohol withdrawal, hydro-electrolyte imbalances, drug abuse and psychiatric disorders. Brain imaging is necessary when the clinical presentation is unusual or sudden, when there are diagnostic doubts, or when the patient does not respond to the usual therapies.

In contrast, the diagnosis of MHE can be made with psychometric testing, both computerized and non-computerized, and electrophysiologic testing, such as electroencephalography (EEG) and evoked related potentials (ERPs) [1]. 

The main goals of treating HE are to manage the acute event, reduce its duration and prevent complications and hospitalizations for recurrence. 

The treatment of type C HE is based on four basic principles: adopt general measures for patients with altered consciousness, identify and treat alternative or coexisting causes of altered consciousness, identify and correct precipitating factors and initiate empirical treatments to reduce ammonium levels.

The most common drugs used to reduce ammonium levels are nonabsorbable disaccharides, such as lactulose, and nonabsorbable antibiotics, such as rifaximin.

Lactulose is considered the standard-of-care for treating HE and preventing recurrent episodes because it reduces ammonium levels through several mechanisms:

- Laxative effect: by creating a hyperosmolar intestinal environment, lactulose accelerates intestinal transit and prevents ammonium absorption in the colon;
- Ammonium ionization: acidification of intestinal contents results in ionization of ammonium, which in this form can no longer diffuse freely across cell membranes;
- Bacterial uptake of ammonium and the beneficial effect on intestinal microbiota: volatile fatty acids released because of lactulose metabolism, are used by bacteria as an energy substrate for proliferation, while ammonium trapped in the colon is used as a source of nitrogen for protein synthesis;
- Reduction of the intestinal production of ammonium: lactulose inhibits the activity of the enzyme glutaminase and the intestinal uptake of glutamine, blocking the subsequent conversion into ammonium;

Among the non-absorbable antibiotics, Rifaximin is the most widely used in this field. It is a semi-synthetic and non-absorbable antibiotic whose mechanism of action consists in modulating the function and composition of the intestinal microbiota by acting on gram+ and gram– enterobacteria. It also presents anti-inflammatory and eubiotic effects. It is generally well tolerated and interactions with other drugs are negligible. It is used for acute treatment and for secondary prophylaxis in case of lactulose failure.

Other possible medications or strategies include polyethylene glycol, branched chain amino acids, probiotics and faecal microbiota transplantation [5].

## 2. Metabolic Alterations in Liver Cirrhosis

The liver is the principal organ responsible for distribution, storage and detoxification of nutrients absorbed from the gastrointestinal tract. 

One of its critical roles includes the metabolism and storage of glycogen as an energy source during fasting periods to maintain glucose homeostasis. Therefore, this organ provides glucose during fasting states and energy to cell types that heavily consume glucose, such as neurons, red blood cells and kidney cells. 

Conversely, when blood glucose level rises, the liver increases glycogen synthesis and suppresses hepatic glycogenolysis and gluconeogenesis [6]. 

Another function of the liver is the production of bile acids; they promote lipid digestion in the small intestine and indirectly participate to lipid and glucose metabolism. 

In addition, the liver is involved in the storage of micronutrients and in the detoxification of products derived from the gastrointestinal tract, primarily ammonia, which is converted to urea through the urea cycle [6]. 

When liver function fails, these functions are severely compromised. 

In cirrhosis, which is the final stage of all chronic liver diseases, major metabolic alterations are observed, and they cause high mortality and reduced quality of life. Several metabolic mechanisms are compromised in patients with liver cirrhosis:

Carbohydrate metabolism: as a major consequence of cirrhosis, the hepatic glycogen storage capacity becomes significantly reduced and this causes abnormalities in energy production. 

Because of the decreased ability to utilize glucose, lipid oxidation and gluconeogenesis are accelerated, leading to a higher incidence of post-prandial hyperglycaemia [6]. This condition is also due to insulin resistance; in fact, a significantly lower insulin sensitivity index after oral tolerance testing and increased pancreatic insulin synthesis have been demonstrated in several studies to compensate the lack of glucose clearance [7]. Up to 70% of cirrhotic patients have some degree of glucose intolerance or insulin resistance, whereas 14–46% have type II diabetes mellitus [8].

Moreover, hyper catabolism and hyperglucagonemia disproportionate to insulin level are observed in cirrhotic patients, with an increased glucagon to insulin ratio [9]. 

Protein metabolism: protein turnover is also enhanced; in fact, amino acids are used as alternative substrates for hepatic gluconeogenesis. Therefore, the body becomes highly catabolic to support the increased protein demand. 

In addition, the use of branched-chain amino acids (BCAAs) such as leucine, isoleucine and valine obtained from muscle catabolism for glutamine synthesis and ammonium clearance, cause an imbalance in plasma amino acid concentrations with predominance of aromatic amino acids (AAA), i.e., tryptophan, phenylalanine and tyrosine [6]. 

A low BCAA to AAA ratio has been shown to be associated with a worse prognosis and risk of developing HE [10]. When this ratio is <3, AAAs cross the blood–brain barrier and act as substrates for the synthesis of false neurotransmitters such as octopamine, tyramine and phenylethanolamine, which displace major neurotransmitters such as dopamine and norepinephrine [7]. 

Unlike healthy subjects in whom these responses to fasting are observed after 2 or 3 days, in cirrhotic patients these changes already occur during the night; in fact, a high catabolic activity of the organism has been demonstrated during this period [11]. This adaptation mechanism is related to the scarcity of glycogen reserves [12]. 

Lipid metabolism: in cirrhotic patients are observed an increased lipolysis and oxidation of non-esterified fatty acids with the aim of obtaining energy from other sources [9]. 

Other alterations: additional metabolic alterations observed in these patients include alteration in appetite-regulating hormones and, in patients with cholestasis, reduction in the pool of bile salts resulting in malabsorption of fats and fat-soluble vitamins [13].

These metabolic changes may have an influence on basal metabolism, although some studies have shown contradictory results. In fact, some studies have shown that up to 34% of patients have a rest energy expenditure (REE) of 120% above the expected value [14], while in others this value is normal in most patients [15].

## 3. Protein-Calorie Malnutrition in Liver Cirrhosis

In addition to known complications such as HE, digestive haemorrhage and ascites, liver cirrhosis is associated with altered nutritional status that can cause severe complications and reduced life expectancy. 

Some consider malnutrition as the most frequent complication of liver cirrhosis. 

Malnutrition is defined as a state of continuous and inadequate oral intake that results in altered nutritional status with significant loss of weight and muscle mass [6].

The best term to define malnutrition in cirrhotic patients is protein-calorie malnutrition, in which both muscle and fat mass are depleted. However, the predominant and later loss of muscle mass in cirrhotic patient suggests that sarcopenia represents the major nutritional deficiency [16]. 

Protein-calorie malnutrition affects 25 to 56% of patients with liver cirrhosis. Although it is more frequent in patients with advanced disease, surprisingly it is present in up to 16% of Child-Pugh class A patients [7]. Thus, even patients with modest disease are at risk of malnutrition. 

However, malnutrition cannot be considered only a consequence of liver disease, because it can accelerate its natural history and negatively affect the patient’s prognosis exacerbating the vicious cycle of anorexia-cachexia [17].

Anorexia is defined as pathological lack of appetite that is not conscious refusal of food, but loss of sense of hunger and desire to eat with a persistent sense of fullness. 

In liver cirrhosis, this condition may be due to various factors such as systemic inflammation, polypharmacotherapy, dyspepsia, maldigestion, malabsorption and social or psychiatric factors.

Anorexia in turn can cause pre-cachexia and cachexia, which is the loss of weight and fat mass, and sarcopenia, which is the loss of muscle mass and function. 

However, cachexia and sarcopenia do not always coexist in liver cirrhosis. For example, inactivity can cause an increase in weight and fat mass and simultaneously a reduction in muscle mass. This condition is known as sarcopenic obesity [15].

The presence of protein-calorie malnutrition is associated with increased complications of liver cirrhosis, such as digestive variceal haemorrhage, HE, hepatorenal syndrome, impaired liver function and regeneration capacity and post-surgical morbidity and mortality. Malnutrition is also an independent predictor of mortality in cirrhotic patients [8]. 

The causes of malnutrition are diverse and include nausea, loss of appetite, alterations in gustatory receptors, reduced oral energy and protein intake, dysgeusia due to zinc or magnesium deficiency, increased basal metabolic rate, unappealing hypoprotein diets, also often restricted in sodium and fluid content, gastroparesis and increased circulating leptin levels [18]. Certain medications, such as diuretics and lactulose, may also be the cause of nutritional deficiencies. Equally, alcohol abuse can cause loss of appetite [16].

In cirrhotic patients, portal hypertension can cause malabsorption that results in changes in intestinal mucosa, such as increased intestinal permeability and consequent protein loss [16]. Finally, in patient with HE, frequent hospitalizations, confusion or excessive sleepiness, may cause a worsening of nutritional status [18].

## 4. Beyond BMI: Sarcopenia, Myosteatosis and Sarcopenic Obesity in Liver Cirrhosis

In cirrhotic patients, the assessment of nutritional status based on BMI may not be reliable because it can be influenced by water retention. In addition, several studies have now ascertained the presence of alterations in body composition, such as sarcopenia and myosteatosis.

Sarcopenia affects 40 to 70% of patients and the pathogenesis appears to be multifactorial. Possible causes include increased plasma levels of myostatin, reduced caloric and protein intake, intestinal malabsorption of nutrients and increased pro-inflammatory cytokines [6]. 

The presence of sarcopenia, which is also associated with impaired muscle function, increases the risk of sepsis and mortality by two- to three-fold [17].

Some evidence has shown that patients with cirrhosis can develop both loss of skeletal muscle and increase in intermuscular and intramuscular fat, a condition called ‘myosteatosis’, characterized by a reduction in radiodensity on CT scans.

This complication is independent of dietary lipid introduction because pathological fat deposition can also be observed in non-obese or even in underweight patients.

Mechanisms of pathological lipid storage within the muscle in cirrhosis have not been identified but may be linked to metabolic aberrations associated with hepatic dysfunction. In fact, elevated skeletal muscle ammonia uptake can promote skeletal muscle mitochondrial dysfunction via diminished lipid oxidation, which results in the accumulation of lipid mediators. 

Sarcopenia and myosteatosis can coexist in cirrhotic patients, although in the study by Ebadi et al. this coexistence affected only 17% of the patient cohort [19].

Several studies have shown that sarcopenia and myosteatosis are associated with poor prognosis and with different complications including HE. 

In fact, the study by Bhanji et al. demonstrated that sarcopenia and myosteatosis were independently associated with OHE risk in patients with liver cirrhosis [20], while in the study by Montano-Loza et al., sarcopenia (HR = 2.00) and myosteatosis (HR = 1.42) were significantly associated with an increased risk of mortality [21]. 

Patients in whom these two conditions coexist, would appear to have the worst prognosis.

A study was recently conducted on the prognostic role of myostatosis in transplanted patients. In this study, the probability of graft and patient survival at five years was significantly worse in the presence of myosteatosis. These patients showed significantly higher all-cause mortality, mostly due to respiratory and septic complications. Surprisingly, sarcopenia was not significantly associated with graft and patient survival [22]. 

In this context, it must be considered that in recent years the number of overweight or obese cirrhotic patients has significantly increased. In spite of what one may think, these patients can be malnourished in the same way as normal weight or overweight patients; therefore, even and especially for them, an early dietary intervention is indicated. 

In fact, a lot of patients with liver cirrhosis tend to be presenting with obesity these days due to changes in lifestyle. 

“Obesity paradox” is a phenomenon in which obese patients seem to have a reduced risk of death compared to people with standard weight; this is because while obesity is associated with increased adipose tissue mass, it is difficult to accurately assess adipose tissue mass with BMI, which includes muscle mass and bone mass in addition to fat. In fact, there are cases of excess visceral fat even when BMI is within the normal range [23]. 

Sarcopenia and obesity are closely related, with a prevalence from 2 to 46%; Montano-Loza et al. reported that patients with sarcopenic obesity had significantly poorer survival compared to the control group (median OS, 22 months) [21] and this condition could compromise the outcome of surgery. In fact, Kobayashi et al. reported that sarcopenic obesity was an independent risk factor for mortality (HR = 2.504, *p* = 0.005) and HCC recurrence (HR = 2.031, *p* = 0.006) after hepatic resection for hepatocellular carcinoma [24].

It is therefore important to look at the emerging clinical determinants of cirrhosis, such as muscle alterations, from a different perspective, in which new factors could add prognostic value to the oldest and most well-established ones, such as MELD.

Indeed, not only the presence of a severe liver disease, or a previous history of minimal/covert HE, or iatrogenic portosystemic shunts, but also sarcopenia, nutritional deficit or spontaneous portosystemic shunts could play a major role.

The attention to muscular alterations could also have a therapeutic implication. In fact, current therapies for HE aim to lower ammonium levels, since its pathogenetic role in cognitive alterations in cirrhotic patients is well known. 

However, the improvement of nutritional status and body composition should be considered an important endpoint in the management of these patients because it could have a preventive and prognostically favourable role.

## 5. Malnutrition in Patients with Hepatic Encephalopathy

The role of nutrition in the pathophysiology of HE has long been hypothesized. Indeed, because HE is a late complication of advanced liver cirrhosis, it is not surprising that cirrhotic patients are susceptible to nutritional disorders. 

Nutrition and HE may influence each other for several reasons [25]. 

Ammonium metabolism is probably the most studied mechanism of nutritional effects on HE, because its production is strongly influenced by diet. Indeed, because most protein substrates for bacterial fermentation originate from dietary protein, it is safe to assume that dietary interventions that affect proteins can have a significant therapeutic impact [6]. 

Skeletal muscle may play a compensatory role in ammonia clearance through glutamine synthase, which metabolizes ammonia into glutamine. Consequently, muscle depletion may favour ammonia accumulation and HE development. 

In fact, approximately 75% of patients who develop HE have moderate-to-severe malnutrition and this condition is associated with an increased risk of mortality. 

In fact, as we recently demonstrated with our group, the presence of sarcopenia (29% vs. 7%, *p* < 0.0001), previous HE (28% vs. 6%, *p* < 0.001) or HE during follow-up (25% vs. 9%, *p* = 0.005) were associated with a higher mortality rate. The co-presence of previous HE and sarcopenia were independently associated with mortality (HR 2.56, *p* = 0.0056, 95% CI 1.3–5) [26]. 

For this reason, improving nutritional status must be considered a key goal to improve cognitive decline in these patients. So, dietary modulation should be considered a valid option when trying to prevent episodes of HE in patients with liver cirrhosis.

The existence of a relationship between HE and nitrogen-containing foods has long been claimed. 

The initial evidence in support of this hypothesis derives from animal experiments conducted in the late 1800s; in particular, in portocaval derived dogs, a meat-based diet caused neurological symptoms, while if dogs were fed with bread and milk or did not lose weight, no neurological complications were observed [12]. 

Based on this evidence, Sherlock et al. first described that, in cirrhotic patients, symptoms of HE could be controlled by a low-protein diet. 

For this reason, the first published studies on HE treatment suggested protein restriction as an effective strategy to reduce plasma ammonium levels and improve HE symptoms and has been the cornerstone treatment of HE since the 1950s. However, clinical benefits on neurological symptoms have not always been observed. 

In fact, an hypoproteic diet in patients with reduced oral nutrient intake and in a hypercatabolic state, can worsen the patient’s nutritional status and prognosis.

## 6. Assessment of Nutritional Status

In 2019, the European Association for the Study of the Liver (EASL) published new guidelines regarding nutrition in patients with chronic liver disease. The aim was to answer a number of questions related to this topic, such as how to recognize nutritional problems and which patients need evaluation, what are the consequences of malnutrition and how to intervene for its correction and prevention. 

These guidelines consider the problem of malnutrition in several areas, including that of obesity and hepatic encephalopathy, and focus attention on sarcopenia and how to intervene to prevent it [1]. 

Acquiring a diagnosis of malnutrition in the early stages of liver cirrhosis can be complicated and there is no consensus on the best method to quantify and classify this condition. Since malnutrition and loss of muscle mass are frequently observed in cirrhosis, a preliminary step in the evaluation of cirrhotic patients is the definition of nutritional status [12]. In fact, it represents the first step to define the pattern of tissue loss and to establish the appropriate treatment strategies. 

The knowledge on the pathophysiology of HE and the evidence of the prognostic impact of sarcopenia, makes the assessment of muscle mass and function a key element in the evaluation of cirrhotic patients. Of these, the most complex techniques for assessing nutritional status require patient collaboration, often high costs and trained personnel, but they are useful for confirming patient’s bedside measurements. 

Screening should be performed in all patients, but especially in those at higher risk of malnutrition such as those with advanced cirrhosis (Child-Pugh class C) and underweight (Body mass index, BMI, <18.5) [27]. 

However, there are some limitations to the definition of nutritional status in cirrhotic patients [6]:

- There are gender differences in body composition and tissue loss characteristics that limit the usefulness of instruments measuring muscle mass and function in women. In fact, the study by Riggio et al. showed that body composition is different between men and women. In particular, in women, fat reserves were more deficient with maintenance of muscle mass. In contrast, in men, the loss of muscle tissue was more evident as observed under stress conditions [28].
- There is no standardized approach to diagnosing and classifying malnutrition;
- Prevalence is affected by aetiology of cirrhosis, being very high in hospitalized patients with alcoholic aetiology;
- Hydrosaline retention makes body weight and body mass index unreliable;
- The value of biochemical markers, such as albumin, are affected by plasma dilution and altered hepatic synthesis;
- More accurate measurements, such as DEXA and dilution techniques, have high costs, are not always available and require specialized personnel.

There are several tools used in clinical practice to assess nutritional status, including those that combine objective and subjective data.

### 6.1. Non-Instrumental Methods

- Food diary: represents a simple tool to obtain information regarding daily food intake [8];
- Objective examination: allows recognition of signs of nutritional deficiency such as loss of muscle mass, loss of subcutaneous fat, dry skin, hair loss and signs of vitamin and micronutrient deficiencies;
- Biochemical parameters: parameters such as albumin, pre-albumin and retinol-binding protein are influenced by the residual capacity of hepatic synthesis, so they are not reliable for the assessment of nutritional status in cirrhotic patients; therefore, the level of total plasma proteins correlates more with severity of hepatopathy than with nutritional status [8].
- Micronutrient dosage: zinc deficiency is extremely common in cirrhotic patients and has a prevalence of 84–96%; it may be due to reduced intestinal absorption or excessive diuretic use; symptoms of deficiency include anorexia, immune system dysfunction and dysgeusia [8]; in addition, because zinc participates in urea detoxification, it may increase the risk of HE.

As known, hyponatremia can cause cognitive decline by acting on astrocyte swelling. Both hypovolaemic and hypervolaemic hyponatremia can occur in patients with cirrhosis. The second one is characterized by an expansion of extracellular fluid volume, with ascites and oedema. 

Splanchnic vasodilation and arterial underfilling play a major role in development of this type of hyponatremia: the opening of porto-systemic collaterals and the synthesis of circulating vasodilators causes the reduction in vascular resistance predominantly in the splanchnic arterial circulation; this condition leads to a decrease in the effective circulatory volume and, in order to restore the effective circulatory volume, the sodium-retaining neurohumoral mechanisms, such as the renin-angiotensin-aldosterone system, sympathetic nervous system and ADH, are activated leading to maximal retention of sodium and water [1,29].

In addition, patients with cirrhosis and ascites usually follow a sodium-deficient diet, which may precipitate neurological symptoms in addition to water retention.

On the contrary, hypovolaemic hyponatremia is characterized by the frequent absence of ascites and oedema as a result of a prolonged negative sodium balance with marked loss of extracellular fluid due to excessive diuretic therapy [1].

- Plasma vitamin dosage: among possible vitamin deficiencies, thiamine deficiency is often encountered, especially in alcoholic aetiology, because of reduced intake, reduced storage and reduced intestinal absorption [7]. Its deficiency causes Wernicke’s encephalopathy, which must be placed in differential diagnosis with HE, so it must always be supplemented.

Vitamin D may also be deficient because of reduced dietary intake, reduced exposure to sunlight, reduced intestinal absorption, deficiency of binding proteins and impaired protein hydroxylation. Rarely, it causes osteomalacia.

A study by Vidot et al. demonstrated that there is a significant correlation between 25-OH vitamin D deficiency and the presence of HE; in fact, with equal disease severity, episodes of HE were more frequent in patients with low vitamin D levels [30]. 

Vitamin B12 and folic acid deficiencies may develop primarily from a loss of hepatic storage in advanced stages of disease. Normal plasma values may not reflect actual tissue levels of them. Symptoms of deficiency include anaemia, glossitis and neuropsychiatric manifestations [8].

Retinol deficiency may be caused by reduced intestinal absorption and hepatic clearance of vitamins. Vitamin A is stored in stellate cells and its deficiency may promote collagen release and fibrosis [31]. Vitamin A deficiency can cause night blindness, photophobia and increased risk of neoplastic complications. In addition, a level <78 umol/L in cirrhotic patients is associated with an increased risk of mortality. However, because high doses of vitamin A are hepatotoxic, supplementation should be conducted with caution [32,33]. In patients with cholestasis or alcoholic aetiology, a deficiency of fat-soluble vitamins may be found [8]

- Creatinine-to-weight ratio: if patient’s renal function is normal, this ratio can be used to estimate muscle mass. In fact, creatinine is almost entirely contained in skeletal and smooth muscle and reduced urinary excretion may be due either to impaired renal function or to reduced muscle mass, but not to impaired hepatic function [33];
- Anthropometric measurements: these are objective methods for assessing patient’s nutritional status. They are rapid, non-invasive and low-cost techniques specifically designed to assess somatic characteristics. However, even these have limitations when applied to cirrhotic patients, especially with HE [25].

Among anthropometric measures, certainly the best known is BMI. Its value can be altered by electrolyte alterations, renal insufficiency and presence of ascites or oedema; therefore, it has a low sensitivity and poor reliability for measuring nutritional status in these subjects [8]. 

The measurement of triceps skin fold thickness (TSF) and mid-arm muscle circumference (MAMC) are less affected by water retention than BMI, although they can be influenced by sex and oedema in the case of upper limb localization [8]. In fact, while the value of MAMC seems to be more compromised in men, the opposite is true for TSF in women. Moreover, obesity may compromise a correct assessment and both measures are subject to some inter-observer variability [25]. 

However, these measurements are easily performed at patient’s bedside, provide an immediate assessment of fat and muscle mass, respectively [6], and are considered one of the best methods for indirect assessment of patient’s nutritional status. 

Several studies have been conducted on these methods. 

A study by Fiore et al. confirmed the reliability of these methods and demonstrated that the percentage of fat mass identified with the skinfold method differed by less than 5% from that obtained with DEXA [34].

A study by Alberino et al. proposed that TSF and MAMC could be included in the Child-Pugh classification to improve its predictive value, although the prognostic accuracy of TSF is lower than that of MAMC [35]. 

Regarding the association with HE, the study by Merli et al. confirmed that the prevalence of this complication among hospitalized patients was higher in patients with low muscle mass, as measured by TSF and MAMC, as well as in those with reduced muscle strength [36].

- Subjective global assessment questionnaire (SGA): this is one of the most widely used methods for hospitalized patients. It is a questionnaire that uses several components of nutritional assessment including objective examination, dietary and clinical history (weight, dietary intake, gastrointestinal symptoms, functional capacity, nutritional demands and metabolic demands), to classify patients according to their degree of malnutrition. It is quick and easy to administer and takes approximately 15 min; it has been validated for nutritional assessment in cirrhotic patient and may provide prognostic information [8].

However, this method has some methodological limitations in patient with HE. In fact, personal information is required, and they are difficult to obtain in patients with impaired cognitive status; moreover, the only anthropometric measure used is body weight, which is often affected by the presence of ascites or oedema; finally, it is poorly sensitive in detecting early stages of malnutrition [6] and in fact the ISHEN guidelines state the possibility of underestimating malnutrition with this method [27].

### 6.2. Instrumental Methods

- Bioimpedance testing: it is a tricompartmental model technique as it identifies the muscle body mass and the non-fat body mass, which is divided into extracellular body mass and cellular body mass or metabolically active tissue.

This is based on the principle that different types of tissue express a specific electrical conductivity, that make them recognizable. In particular, electrical conduction is faster through water and slower through adipose tissue, due to the resistance imposed by fat deposits. 

It is a non-invasive, safe, simple to perform, inexpensive and sensitive method to obtain information about the subject’s body composition and also prognostic information. However, it has some limitations in patients with electrolyte disturbances [8]. 

The measurement is performed by placing a pair of electrodes on the back of the hand and another pair on the back of the subject’s foot [tetra-polar hand–foot technique), which are then connected to the measuring instrument. Then an imperceptible alternating current of very low intensity (800 µA) and high frequency (50 KHz) is passed through the electrodes. The software then transforms the detected electrical measurements into clinical data, based on algorithms that take into account the reference values, the anthropometric measurements (weight and height), his age and sex. 

The values obtained from these measurements are phase angle (PA) and body cell mass (BCM).

The latter estimates the body cellular elements and has been considered one of the best nutritional parameters to assess metabolic pathways such as protein turnover and energy expenditure [25]. Phase angle (PA) represents a measure of strength and muscle mass, and this value is reduced in advanced liver cirrhosis. 

BCM identifies muscle body mass, and this is reduced in protein-calorie malnutrition and advanced stages of cirrhosis [25].

- Handgrip strength: this is an instrument used to assess muscle strength. It is one of the most sensitive methods for measuring nutritional status and has been shown to predict prognosis in patients with advanced liver disease [6].

In the study by Alvares-da-Silva et al., the ability of handgrip to detect malnutrition was superior to SGA; moreover, this method was the only one able to predict the incidence of significant complications of cirrhosis at one year in malnourished cirrhotic patients [37]. 

Regarding the association with HE, in the study by Merli et al., the prevalence of HE, both minimal and overt, was higher in patients with impaired muscle strength measured by this method, suggesting a correlation between muscle function and HE [36].

However, this method has some limitations, especially in relation to the different body composition between men and women. 

In fact, according to the ISHEN consensus, muscle function is associated with muscle mass only in males; therefore, dynamometry would not be a reliable tool for measuring nutritional status in women [27].

- DEXA: this examination has received particular attention because it is widely used to validate body composition results obtained with other methods. The procedure is based on measuring body composition according to a model that divides body elements into bone, fat, muscle mass and body free mass based on the passage of photon. In addition, compared to other techniques, radiation exposure is minimal [25].

Thus, DEXA allows the assessment of muscle mass and fat mass, with good correlation with both bioimpedance and anthropometric measurements [25]. 

With DEXA we can obtain the muscle mass to height squared ratio, called fat-free mass index (FFMI). In the study by Kalaitzakis et al. in cirrhotic patients awaiting liver transplantation, this index was found to be an independent predictor of risk of HE [38]. 

DEXA can also be used to calculate the appendicular muscle mass index (AMMI), which is obtained by dividing the appendicular muscle mass of the four limbs (free of fat and bone tissue) by height squared [25]. This index provides a more accurate estimate of muscle mass because it does not use bone density, which is affected by age, ethnicity and medications. It also excludes the trunk, which is often involved in water retention in cirrhotic patients [25]. 

One of the major advantages of DEXA is the high reproducibility of measurements. In addition, it also allows measurement of bone density, and this is an important finding to obtain, because it is often reduced in cirrhotic patients and can increase the risk of fractures [25]. 

Nevertheless, a possible disadvantage is the water imbalance that can alter the passage of X-rays [25].

- Indirect calorimetry: is used to define REE by measuring oxygen consumption and carbon dioxide production. The patient is considered hypermetabolic if the REE is 10–20% higher than the reference value. However, this method is expensive, available only in some centres and may be affected by the presence of ascites [8].
- Computed tomography scan: recently there has been a growing interest in the use of CT scan for the assessment of muscle mass loss and the presence of porto-systemic shunts, that may favour the development of HE in cirrhotic patients.

Measurements are made in a single image at the level of the intervertebral disc between the third and the fourth lumbar vertebra, discriminating muscle tissue from other tissues by density limits. The 35 Hounsfield (HU) limit is used to discriminate muscle tissue from fat, whereas the 150 HU limit is used to discriminate it from bone [25]. 

The skeletal muscle index (SMI) is obtained from muscle area to height squared ratio; sarcopenia is defined when this value is <39 cm^2^m^2^ and 50 cm^2^/m^2^ in women and men, respectively [25]. 

Sarcopenia, defined according to this method, is significantly correlated with mortality [30]. The limitations of this method are certainly the high cost and the exposure to ionizing radiation [25].

- Assessment of global physical performance: up-and-go test, six minutes’ walk test.

As evident, for reasons of sensitivity, specificity, availability and cost, there is no ideal method for nutritional assessment of cirrhotic patients, especially with HE, since the degree of HE may condition the use of certain methods and compliance of patients. 

Probably the best approach, as also recommended by the European Society for Enteral and Parenteral Nutrition (ESPEN) guidelines, is the multiparametric one. Thus, as an initial assessment, the use of indirect measures such as the SGA scale and anthropometric assessments is recommended, since they are sensitive and adequate to identify subjects at risk of malnutrition. After identifying malnourished subjects, the use of quantitative methods, such as BIA, is recommended as they are more accurate in classifying patients [39].

**Figure 1:**
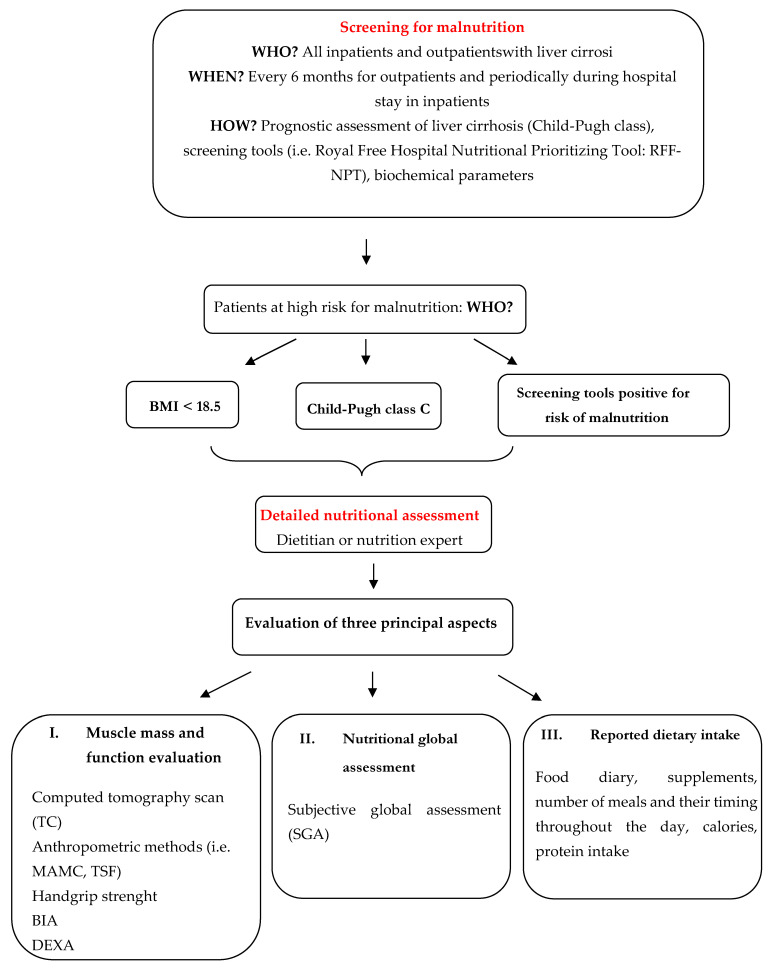
Diagnostic algorithm for nutritional status evaluation in patients with liver cirrhosis.

Figure 1 illustrates the diagnostic algorithm for evaluating the nutritional status in cirrhotic patients.

## 7. Optimization of Nutritional Status

The quality of evidence supporting dietary interventions in liver cirrhosis is poor and limited and those conducted in patients with HE are even more limited. Moreover, studies conducted in this field are often short-lived. 

The multidisciplinary approach is fundamental. In fact, dieticians are not only trained in the aspect of nutritional assessment, but also have the ability to identify nutritional problems and provide appropriate dietary counselling tailored to patients [6]. In contrast, few physicians receive significant training or have the time to perform a comprehensive nutritional assessment; so, they risk underestimating this aspect [6].

Diet, although a key aspect in clinical management of cirrhotic patients, is often underestimated. In fact, in a retrospective study by Huynh et al., only 57% of hospitalized patients received a formal nutritional assessment at time of admission and of these, 56% were judged malnourished [40]. 

The importance of nutritional aspect in cirrhotic patients is confirmed by the study of Iwasa et al. who demonstrated that an early dietary intervention performed with a multidisciplinary approach in patients with cirrhosis was able to improve survival and quality of life [41]. 

Therefore, it seems essential to take care of nutritional aspect of cirrhotic patients as for any other complication of cirrhosis [27]. 

A relevant problem concerns the obesity and metabolic comorbidities of patients with post-NASH cirrhosis, in which patients often appear overweight or obese and in which it is necessary to recommend low-calorie diet and weight loss. 

However, in these patients, in the absence of counselling and a clear-cut specialist, the risk of incurring in sarcopenia is high, despite the underlying obesity. A study by Berzigotti et al., showed that a low-calorie diet, high protein content and moderate physical activity were safe and effective in weight loss if adequately monitored [42]. 

The goals of nutritional intervention are to improve protein-calorie malnutrition, ensure adequate nutrient intake, achieve a positive nitrogen balance and avoid hepatotoxic agents and muscle loss [8]. Other goals are to avoid progression of liver failure and to manage complications arising from the disease [6]. 

As suggested by the ISHEN guidelines, the feeding of cirrhotic patients with HE should not be different from that of cirrhotic patients without HE. In fact, the recommendations are equivalent [27]. What should be avoided are dietary restrictions, which appear harmful, counterproductive and can worsen the protein breakdown.

Strategies that can be implemented to improve the nutritional status of the cirrhotic patient involve several areas.

Table 1 summarizes the dietary recommendations for cirrhotic patients, while Table 2 illustrates the main studies published on this topic.

### 7.1. Caloric Requirement 

Cirrhotic patients who are not malnourished and in the absence of stressful conditions should have 30 Kcal/Kg/day; however, energy demand should be gradually increased to 35–40 Kcal/Kg/day under stressful conditions (e.g., bleeding, infections or surgery) or in malnourished subjects to achieve the state of anabolism [7]. 

The study by Maharshi et al. conducted in India demonstrated that an appropriate nutritional regimen with adequate calorie intake for 6 months in patients with minimal HE (MHE) was effective in treating this condition and especially in preventing progression to overt HE [46]. 

Obviously, caloric excess should also be avoided because it has a detrimental effect on lipiogenesis and thus on residual liver function [8]. 

These recommendations do not apply to obese patients. In fact, in this case the energy intake should be moderately reduced to 20–25 Kcal/Kg/day, avoiding excessive restriction that could be responsible for loss of muscle mass. Energy reduction should concern lipids and carbohydrates, while protein intake should be preserved. In addition to diet, moderate physical activity should be recommended [12]. 

In these patients, the increase in number of daily meals to four–six should also be encouraged, introducing snacks in the mid-morning, mid-afternoon and before bedtime to prevent gluconeogenesis, since this can cause sarcopenia, increased ammonium production and therefore HE. 

Confirming this, the study by Swart et al. showed that consuming four–six meals per day resulted in a positive nitrogen balance, compared to three meals per day [41].

The evening snack should contain complex carbohydrates to ensure slow glucose absorption and should be high in calories (at least 50 g of carbohydrate) [58]. 

Recently, an evening snack containing BCAAs has been shown to be effective in preserving muscle mass, nutritional status and preventing HE episodes, infections and mortality [39]. Indeed, while overnight BCAAs are preferentially used for protein synthesis, during the day they are primarily used as an energy resource [8]. 

### 7.2. Protein

In cirrhotic and HE patients, the goal of maintaining adequate protein intake is to improve nitrogen balance and prevent sarcopenia. 

Prevention of sarcopenia may be advantageous especially in patients with HE, since muscle is known to represent an alternative site for ammonium detoxification. 

In fact, several studies have examined the effect of protein diet on changes in cognitive status in patients with HE, confirming its undoubtedly beneficial role [17]. In contrast, even a transient protein restriction has not shown any benefit in patients with HE. 

Contrary to what was originally thought about the role of protein in encephalopathic patients, in 1997 the ESPEN published new guidelines recommending an adequate protein intake in patients with liver disease, possibly around 1–1.5 g/kg/day, depending on the degree of decompensation and accordingly with renal function. 

In case of intolerance to dietary protein, the same guidelines suggested to administer 0.5 g/Kg/day of protein and to supplement the rest with BCAAs to ensure the achievement of an adequate daily protein intake [39]. This dosage is supported by numerous studies that confirm the achievement of positive nitrogen balance by supplementation. 

In support of this evidence, the study of Cordoba et al. showed that a diet with normal protein content, being metabolically more adequate, could be safely administered to patients with HE while a low protein content diet did not confer any benefit [50].

The effect of protein feeding is related not only to the amount of protein intake, but also to the timing of intake. In fact, night-time supplementation is associated with greater gains in muscle mass than daytime supplementation [6]. 

There is also a strong debate about the origin of dietary protein intake. 

Some studies showed a significant decrease in ammonium levels by replacing meat with dairy products [6]. 

Among proteins, plant proteins contained in fruits, vegetables, cereals and legumes seem to be a good choice to increase dietary protein intake [59], also because they are often better tolerated than animal proteins [60]. 

In this regard, the study by Bianchi et al. demonstrated the superiority of plant proteins over animal proteins in association with lactulose in reducing ammonium levels and improving neurological symptoms in a small group of patients with overt HE [51].

In fact, ISHEN guidelines recommend, in the cirrhotic patient, to prefer vegetable and dairy proteins over meat and fish [27]. 

The reasons why diet rich in vegetable protein and low in animal protein results in improved HE is not clearly known. There are a few possibilities:

- The lower ratio of sulphur amino acids, such as methionine and cysteine, to BCAAs [59];
- The reduced formation of mercaptans from sulphur amino acids fermentation, which appear to be involved in the genesis of HE along with ammonium [13];
- The significant increase in fermentable fibre. In fact, levels of protein fermentation end products, such as ammonium and phenols, are significantly reduced when dietary fibre intake is increased. This occurs because increased fermentation of carbohydrates by colonic bacteria results in increased nitrogen utilization by bacteria and in pH reduction, which favours excretion of ammonium over its absorption [61].
- The increased clearance and reduced intestinal absorption of nitrogen products as a result of reduced intestinal transit time due to the mass-forming effect of fibres [17].
- The modulation of intestinal microbiota [62]. In liver cirrhosis, the prevalence of potentially pathogenic bacteria such as Enterobacteriaceae and Streptococcaceae, and the deficiency of beneficial populations such as Lachnospiraceae, may impact the prognosis of patients with cirrhosis [63].

The microbiota is also modified in patients with HE. In fact, high levels of Alcaligenaceae, Enterobacteriaceae and Fusobacteriaceae and low levels of Ruminococcaceae and Lachnospiraceae have been found in these patients compared with healthy controls and cirrhotic patients without HE [64]. 

In light of this evidence, it could be thought that the beneficial effect of vegetable proteins is actually related mainly to the high fibre content. 

The effect of fermentable fibre, could in this case recall that of lactulose that, once fermented, traps ammonium in the nonabsorbable form NH4 and allows its elimination with the stool [6]. 

Therefore, a rational dietary intervention might be to manipulate the fermentable carbohydrate/protein ratio. This would provide an effect similar to that of lactulose while still ensuring adequate protein intake [6]. 

It is clear that an adequate nutritional intervention cannot be generalized, but must be adapted case by case according to patient, his nutritional status and gastrointestinal tolerance to fermentable fibres [6].

### 7.3. Protein Supplementation

The availability of BCAAs in cirrhotic patients is reduced and this can impair the ammonium conversion to glutamine in skeletal muscle, with deleterious effects on its elimination. 

A review of 16 randomized trials that compared BCAAs, administered orally or intravenous, vs. placebo, no intervention, diet, neomycin or lactulose, revealed that BCAAs have beneficial effects on symptoms and signs of HE, without affecting mortality or quality of life [64]. 

For oral and long-term supplementation with BCAAs, the recommended daily dosage is 0.25 g/Kg/day, which demonstrated beneficial effects on nutrition and reduction in recurrence and symptoms of HE [65]. 

However, some studies have not demonstrated these clear benefits, so ESPEN guidelines do not currently recommend its regular use [39]. 

On nutritional basis, BCAAs have been shown to increase energy and protein intake, reduce anorexia and improve both albumin levels and nitrogen balance. In addiction, they improve the nutritional status of cirrhotic patients by counteracting protein loss and promoting protein synthesis; finally, BCAAs can enhance the innate and adaptive immune response [66]. 

However, a barrier to this supplementation remains patient compliance, which is often reduced due to the poor palatability of the products, the frequency of daily intake and possible associated gastrointestinal symptoms such as abdominal distension and diarrhoea [28]. A viable alternative could be to obtain the same benefits from BCAA-rich foods.

### 7.4. Carbohydrates and Lipids

The literature regarding the role of carbohydrates and lipids in the management of malnutrition is less than that of proteins; so, there are no particular recommendations in cirrhotic patients with HE. 

It is known that complex carbohydrates are useful in delaying the switch to the fasting phase of metabolism, which causes increased amino acid utilization and renal ammoniagenesis [12]. 

The main concern with carbohydrate intake relates to hyperinsulinemia and diabetes, which are seen in approximately 40% of cirrhotic patients. 

However, from the available data, a diet rich in carbohydrates, especially if complex and with low glycaemic index, would not seem to worsen glycaemic control in cirrhotic patients and indeed it would represent an additional source of energy [6]. 

In this regard, the study by Schulte-Frohlinde et al. showed that a diet rich in proteins and carbohydrates resulted in a greater increase in plasma insulin levels than one rich in protein. However, when a low-carbohydrate diet of 20% of daily calories was subsequently instituted, no changes in plasma insulin levels, blood glucose or BCAA/AAAs were observed compared with a high-carbohydrate diet of 60% [67]. 

In general, carbohydrates should therefore represent the mainstay of the diet in the cirrhotic, amounting to approximately 50–60% of non-protein energy requirements [8]. 

Relative to lipids, these can be freely administered to cirrhotic patients. Indeed, nuts such as almonds, hazelnuts and peanuts are highly caloric and rich in lipids and therefore may be useful in cachectic patients with reduced appetite. 

Conversely, lipid reduction should be recommended in obese patients [12].

### 7.5. Fibres, Vitamins and Micronutrients

As previously hypothesized, the beneficial effect of vegetables in patients with liver cirrhosis may actually be due to their high fibre content. 

Therefore, in light of their prebiotic and laxative effect, consumption of fibre from fruits and vegetables should be encouraged in cirrhotic patients with HE, amounting to a daily intake of 25–45 g/day, where tolerated. In obese patients, increased fibre consumption may also be beneficial to achieve early satiety. 

Likewise, cirrhotic patients with HE should be encouraged to consume milk-derived products, such as yogurt, in light of their probiotic effect and modulation of the intestinal microbiota [12]. 

Confirming this evidence, the study by Gheorghe et al. showed that a high-calorie, high-protein diet rich in vegetables and dairy products administered for 14 days to patients with HE in addition to standard therapy was able to determine an improvement in cognitive status in up to 80% of subjects [48].

Relative to vitamins, their deficiency may be responsible for neuropsychiatric symptoms that may overlap with HE symptoms or make their differential diagnosis difficult. Therefore, brief vitamin supplementation is recommended in patients with HE, paying attention to any deficiencies that may develop during follow up [12]. 

Regarding vitamin D supplementation, in cirrhotic patients it is indicated for levels <30 ng/mL by administration of 5000 IU/day of vitamin D3 or 50,000 IU/week of vitamin D2 or D3 for three months. This would also seem to have a favourable impact on survival by enhancing immune defences against viruses and bacteria [7]. 

Finally, in patients with cirrhosis and HE, the hydro-electrolyte balance should always be monitored. Prompt recognition of micronutrient deficiency is crucial because the use of nutritional supplements has been shown to be associated with a reduction in infection risk and in-hospital mortality, as well as with an improvement in liver function [8]. 

Relative to zinc deficiency, which is extremely frequent in cirrhotic patients, the beneficial effects of supplementation on neurologic symptoms are still debated and would appear to be limited. In any case, the deficiency should always be corrected, as good clinical practice, at an oral dosage of 600 mg/day during the treatment of underlying HE [7]. 

Another deficiency that may be encountered in the patient with cirrhosis and HE is sodium deficiency. Management of hyponatremia is challenging for both physicians and patients. 

Hypovolemic hyponatremia should be treated with fluid resuscitation to restore the circulatory volume and withdrawal of the precipitating factor, usually diuretic therapy. 

On the contrary, hypervolemic hyponatremia in cirrhosis is ideally managed with fluid restriction and measures to enhance the renal solute-free water excretion to a level sufficient to induce a negative water balance. Hypertonic saline is indicated in symptomatic patients who are intolerant or unresponsive to free water restriction and in those with profound hyponatremia (<110 mEq/L). In this case, correction of hyponatremia should be conducted slowly to prevent central pontine myelinosis. In general, although a low-sodium diet (<2 g) is recommended in cirrhotic patients with ascites, daily sodium intake should be >60 mmol [1.38 g) to prevent palatability problems and consequent reduction in dietary intake [27]. However, compliance with fluid restriction is usually poor in these patients and conventional therapy is frequently inefficacious because the progression of cirrhosis and ascites leads to impairment of the kidneys to eliminate solute-free water and, while fluid restriction is helpful in preventing a further decrease in serum sodium concentration, it is rarely effective in improving it.

One option could be the use of drugs that act on the hormonal mechanisms underlying hyponatremia; vaptans selectively block arginine-vasopressin hormone V2 receptors in major collector duct cells; however, their use is currently recommended only in an experimental setting [1]. 

Finally, the pragmatic approach in cirrhotic patients, suggested by the ESPEN guidelines, is a brief supplementation of vitamins and micronutrients during the first two weeks of nutritional support, because assessment of deficiency of each micronutrient would require high costs and delays in initiating supplementation [39]. 

### 7.6. Parenteral Nutrition

This represents a second choice to enteral nutrition, but should be initiated when a patient cannot be fed orally or parenterally or when an adequate caloric goal is not achieved. The use of parenteral nutrition, possibly enriched with BCAAs, is indicated in patients with HE who appear comatose because of risk of inhalation, or when enteral nutrition is not possible. It should be initiated in cases of non-functioning gastrointestinal tract, intestinal obstruction, unprotected airway, intolerance to enteral nutrition or when the fasting period exceeds 72 h [8]. 

The recommended caloric requirement is 35 Kcal/Kg/day with 1.2 g/Kg/day of protein, which can be increased to 1.5 in severely malnourished patients or those under severe stress [55]. 

However, enteral nutrition is always preferable to parenteral nutrition because of risk of infection, water overload and cholestasis [7].

### 7.7. Physical Exercise

One thing to keep in mind is the role of physical exercise. In fact, several studies have shown that a combination of diet, dietary supplements and exercise can lead to improved muscle mass in cirrhotic patients. 

In fact, exercise is known to have a beneficial effect on muscle mass in patients with chronic disease [62]. 

The prospective study by Zenith et al. showed that eight weeks of controlled aerobic exercise in patients with Child-Pugh Class A and B liver cirrhosis, was able to increase muscle mass and reduce fatigue, without experiencing any adverse events [57]. 

As previously mentioned, the improvement of muscle mass can have a positive effect on risk of developing HE.

## 8. Conclusions

HE is a frequent complication and one of the most debilitating clinical manifestations of liver disease, associated with decreased survival and a high risk of recurrence. 

Nitrogen metabolism plays a key role in the pathogenesis of HE in patients with liver cirrhosis and therefore its modulation may play a key role in the treatment of this complication. 

One of the first steps in the evaluation of patients with liver cirrhosis, especially with HE, is the assessment of nutritional status. However, this is often problematic because it is conditioned by different body composition between men and women, the cost and availability of some methods, the lack of a standardized evaluation criterion and the need to have the cooperation of patients. This last point is essential in some evaluations, but in patients with altered cognitive status this goal cannot always be achieved. 

Dietary control represents a valid tool to improve the nutritional status and prognosis. In particular, it can be useful to prevent sarcopenia, which is included among the “new precipitants” of HE; in fact, skeletal muscle is involved in the detoxification of ammonium, activating the enzyme glutamine synthase that catalyses the condensation of ammonium with glutamate to form glutamine. 

Despite the impact of nutritional aspects on patient’s prognosis, this field is often neglected or addressed only in stages in which malnutrition is frankly evident. 

Therefore, it would be essential to manage patients in a multidisciplinary team involving a specialized dietician and address this aspect even in the early stages of the disease, in order to prevent the appearance of sarcopenia and potentially other complications of liver cirrhosis such as HE.

## Figures and Tables

**Table 1:** Dietary recommendations for patients with cirrhosis.

	Normal		Moderate Malnutrition		Severe Malnutrition	
BMI	<30	>30	<30	>30	<30	>30
Caloric intake (kcal/die)	35–40	20–35	35–40	20–35	35–40	20–35
Carbohydrate intake (%)	50–60%					
Protein intake (g/die)	1.2–1.5	1–1.5	1.2–1.5			
Number of meals/die	4–6 meals					
Bedtime snacks	High in calories (at least 50 g of complex carbohydrate)					
Protein source	Vegetables and dairy products					
Fibre (g/die)	25–45 g					
Vitamin and micronutrients	Correction of deficiency as good clinical practice					

**Table 2:** Published studies on dietary interventions and exercise in patients with liver cirrhosis.

Target	Author/Year	Study Design	N. Patients	Intervention	Comparison	Duration	Main Results
Number of meals/late snack	Swart et al., 1989 [42]	Randomised crossover	*n* = 9 cirrhotic patients	4–6 meals/die	Three meals/die	2 periods of consecutive five days.	4–6 meals/die resulted in more positive nitrogen balances than three meals/die.
	Plank et al., 2008 [43]	Randomized	*n* = 103 cirrhotic patients	Night-time supplementary nutrition (*n* = 52)	Daytime supplementary nutrition (*n* = 51)	12 months	A night-time snack resulted in a total body protein accretion sustained over 12 months (equivalent to about 2 kg of lean tissue).
	Verboeket-van de Venne, 1995 [11]	Randomized crossover	*n* = 8 cirrhotic patients and 23 healthy subjects (controls)	4–7 meals/die (“nibbling pattern”)	2 large meals (“gorging pattern”)	Two periods of 2 consecutive days.	The “gorging pattern” had greater fluctuations in respiratory quotient and higher nocturnal protein oxidation than in the daytime in both groups, reflecting a higher oxidation ratio of fat to carbohydrate compatible with a more catabolic state.
	Nakaya et al., 2007 [44]	Randomized	*n* = 48 cirrhotic patients	Late-evening supplementation with BCAA-enriched nutrient mixture (= 25)	Late-evening supplementation with ordinary food (*n* = 23)	3 months	BCAA supplementation significantly improved serum albumin level, nitrogen balance and respiratory quotient than ordinary food.
Caloric and protein intake	Manguso et al., 2005 [45]	Randomized	*n* = 90 cirrhotic patients	Controlled diet (*n* = 45)	Spontaneous diet (*n* = 45)	3 months	The controlled diet caused an increase in MAMC, serum albumin and creatinine-height index
	Maharshi et al., 2016 [46]	Randomized	*n* = 120 cirrhotic patients with MHE	Nutritional therapy (30–35 kcal/kg/die and 1.0–1.5 g vegetable protein/kg/die)	No nutritional therapy	6 months	A higher proportion of patients in the nutritional therapy group reversed MHE; nutritional therapy increased PHES and HRQOL and reduced OHE incidence.
	Kato et al., 2013 [47]	Prospective	*n* = 19 cirrhotic patients with MHE	Nutritional consultation (30–35 Kcal/Kg/die and 1–1.5 g/Kg/die of protein)	-	8 weeks	The MHE scores significantly improved at 8 weeks.
	Gheorghe et al., 2005 [48]	Prospective	*n* = 153 cirrhotic patients with OHE	High caloric and high protein diet (vegetable and dairy products)	-	1 year	Almost 80% of patients improved their mental status; high protein diet significantly reduced ammonia level.
	Hirsch et al., 1993 [49]	Randomized	*n* = 51 patients with decompensated alcoholic cirrhosis	Oral nutrition support (1000 Kcal, 34 g protein) (*n* = 26)	One placebo capsule (*n* = 25)		Oral nutrition support significantly improved nutritional status, MAMC, serum albumin and handgrip strength than placebo.
	Cordoba et al., 2004 [50]	Randomized	*n* = 30 cirrhotic patients with acute HE	Normal protein diet (*n* = 10)	Low protein diet (*n* = 10)	14 days	The outcome of HE was not significantly different between both groups.
Protein source/ type of protein	Bianchi et al., 1993 [51]	Crossover randomized	*n* = 8 cirrhotic patients with chronic HE in therapy with lactulose	Diet containing vegetal proteins (50 g)	Isocaloric, isonitrogenous diets containing animal protein (50 g)	2 consecutive periods of 7 days	The vegetable protein diet improved ammonia level, insulin and nitrogen balance, and clinical grading of HE. Psycometric tests improved significantly but remained abnormal
	Ruiz-Margáin et al., 2017 [52]	Randomized	*n* = 72 Cirrhotic patients	High protein and high-fibre diet with BCAA (protein: 1.2 g/Kg/die, fibre 30 g, BCAA 110 g) (*n* = 37)	High protein, high-fibre diet and no BCAA (*n* = 35)	6 months	BCAA supplementation increased muscle mass. No significant changes in PHES or CFF score resulted in both groups (no development of HE).
	Horst et al., 1984 [53]	Randomized	*n* = 37 cirrhotic patients with recurrent HE	20 g of dietary protein for 1 week, after which BCAA were added weekly to obtain a protein intake of 80 g/die (*n* = 14)	20 g of dietary protein for 1 week, after which 20 g of proteins were added weekly to obtain a protein intake of 80 g/die (*n* = 12)		BCAA supplementation significantly reduced HE recurrence and improved mental status grade and asterixis.
	Uribe et al., 1982 [54]	Crossover randomized single blind	*n* = 10 cirrhotic patients with chronic HE	40 g/die of vegetable protein (high fibre diet, low methionine and low aromatic amino acids) and 80 g/die of vegetable protein (rich in BCAA and fibre, with same amount of sulfurated amino acids).	40 g/die of meat protein plus neomycin-milk of magnesia	3 consecutive periods of 2 weeks	After 2 weeks, patients on vegetarian diets performed the NCT more quickly than meat diet. Patients treated with the 80 g/day vegetable diet improved EEG.
	De Brujin et al., 1983 [55]	Randomized crossover	*n* = 8 cirrhotic patients with MHE	60 g/die of vegetable diet (second and fourth week)	60 g/die of a mix diet with 1:1 ratio of vegetable and meat diet (first, third and fifth week)	5 consecutive periods of 1 week.	During the vegetable diet, the nitrogen balance tended to be more positive, but without changes in neurological status or ammonia level.
	Keshavarzian et al., 1984 [56]	Crossover randomized	*n* = 6 cirrhotic patients with chronic HE on lactulose therapy	80 g vegetable-supplemented diet (3:5 ratio of animal and vegetable protein)	40 g protein conventional diet (3:1 ratio of animal and vegetable protein)	2 consecutive periods of 5 days.	After 10 days, patients treated with a vegetable diet showed clinical improvement and amelioration of EEG.
Physical exercise	Zenith et al., 2014 [57]	Randomized	*n* = 20	exercise training (*n* = 10)	usual care (*n* = 10)	8 weeks	Aerobic exercise increased peak VO2 and muscle mass and reduced fatigue in cirrhotic patients.

## Data Availability

Data sharing is not applicable to this article as no new data were created or analized in this study.
